# Comparative Efficacy and Safety of Tirzepatide versus Semaglutide: A Systematic Review and Meta-Analysis with Cardiometabolic Implications

**DOI:** 10.3126/nje.v16i1.92323

**Published:** 2026-04-02

**Authors:** Azhar Hafiz Baba, Rameez Akhtar, Anurag Rawat, Huzaifa Bin Afzaal, Affan Ul Haq, Mazen Bin Khalid, Muhammad Anas Ghazi, Jahanzaib Awan, Pershan Kumar, Yamini Saraswathi Gurram, Muneeb Khawar

**Affiliations:** 1 University Hospitals Bristol and Weston NHS Foundation Trust, Bristol, United Kingdom; 2 Luqman International Hospital, Swat, Pakistan; 3 Himalayan Institute of Medical Sciences, Dehradun, India; 4 Rai Medical College, Sargodha, Pakistan; 5 King Edward Medical University, Lahore, Pakistan; 6 Services Hospital, Lahore, Pakistan; 7 North Manchester General Hospital, Manchester, United Kingdom; 8 Islamic International Medical College, Rawalpindi, Pakistan; 9 Jinnah Medical and Dental College, Karachi, Pakistan; 10 Dr. Pinnamaneni Siddhartha Institute of Medical Sciences and Research Foundation, Vijayawada, India

**Keywords:** Tirzepatide, Semaglutide, Obesity, Cardiovascular outcomes, Diabetes

## Abstract

**Background:**

Obesity and type 2 diabetes are significant global health problems. Tirzepatide, a dual GIP/GLP-1 receptor agonist, and semaglutide, a selective GLP-1 receptor agonist, are the top incretin therapy options, but their relative effectiveness and safety based on direct head- to -head studies have not been pooled. This meta-analysis aims to compare the efficacy and safety of tirzepatide versus semaglutide.

**Methods:**

PubMed, Embase, and ScienceDirect were searched up to February 2026 to find the articles. Mean differences (MD) and risk ratios (RR) were pooled using a random-effects model and heterogeneity was measured using I^2^.

**Results:**

Three studies were included. Tirzepatide caused significantly greater reduction in weight than semaglutide (pooled MD = −5.19 kg, 95% CI: −7.96 to −2.42; p = 0.0002; I^2^ = 88.5%) and increased the possibility of ≥10% weight loss (pooled RR = 1.50, 95% CI: 1.15–1.96; p = 0.0069; I^2^ = 86.3%). No significant difference was observed in any adverse events, or gastrointestinal events; however, there was a greater number of serious adverse events with tirzepatide (RR = 1.83, 95% CI: 1.18-2.85; p = 0.007).

**Conclusion:**

Tirzepatide can provide better weight loss than semaglutide with relatively similar safety, except that serious adverse events were more frequent. Studies with long follow-up are required to confirm the cardiovascular safety.

## Introduction

Obesity is a relapsing chronic disease that has become an epidemic across the world. By 2022, there were more than 890 million adults with obesity living throughout the world, and this number increased more than twofold since 1990 [1]. The condition is a proven risk factor of type 2 diabetes mellitus (T2DM), cardiovascular disease, and various cancers, and it causes a significant fraction of one in eight non-communicable disease-related deaths [2]. In spite of the existence of lifestyle interventions and bariatric surgery, effective pharmacological options that can yield long-lasting and clinically significant weight reduction have long been lacking.

Glucagon-like peptide-1 receptor agonists (GLP-1 RAs) have revolutionized the management of obesity and T2DM. The mechanisms of action of these agents include the stimulation of insulin secretion, inhibition of glucagon secretion, delayed gastric emptying, and inhibition of appetite [3]. Clinical trials have demonstrated semaglutide, a selective GLP-1 RA, to reduce HbA1c by up to 1.8 percentage points and body weight by up to 15 percent, and has been approved both as T2DM and chronic weight management [4,5]. Recently, tirzepatide, a first-in-class dual glucose-dependent insulinotropic polypeptide (GIP) and GLP-1 receptor agonist was approved by the FDA to treat T2DM in 2022 and to treat obesity in 2023 [6]. Simultaneously acting on incretin pathways, tirzepatide demonstrated strong glucose-reducing and weight-lowering effects that seem to be more impressive than those of GLP-1 RAs on their own in preliminary studies [7].

The initial head-to-head RCT that compared tirzepatide to semaglutide on metformin-controlled T2DM patients was the SURPASS-2 trial, which described that tirzepatide at all three doses (5, 10 and 15 mg) was better than semaglutide 1 mg in both reducing HbA1c and weight loss after 40 weeks [8]. Then, SURMOUNT-5 assessed the two agents in obese adults without diabetes after 72 weeks and found that tirzepatide had a mean body weight loss of 20.2 percent compared to semaglutide, which had 13.7 percent [9]. Although these individual trials prefer tirzepatide, the relative safety profile of the two drugs in pooled data has not been well defined.

Up to now, no meta-analysis has combined data of head-to-head RCT of tirzepatide versus semaglutide with both efficacy and all adverse events. Current reviews have either been limited to indirect comparisons provided by network meta-analyses or have pooled trial data provided by placebo trials or other active comparators [10]. This systematic review and meta-analysis will address that gap by providing a synthesis of all available direct-comparison RCTs to determine the relative efficacy and safety of tirzepatide versus semaglutide in reducing weight, glycaemic control, and adverse event rates.

## Methodology

This meta-analysis and systematic review were performed according to the Preferred Reporting Items of Systematic Reviews and Meta-Analyses (PRISMA) 2020 guidelines [11]. The protocol of study was registered at PROSPERO (CRD420261346139).

### Search Strategy

A comprehensive search was conducted in PubMed, Embase, and Cochrane Central Register of Controlled Trials (CENTRAL) since the beginning of the databases up to February 2026. The search involved the use of MeSH terms and free-text keywords: tirzepatide AND semaglutide AND (randomized controlled trial OR clinical trial). Additional records were manually screened in reference lists of included studies and relevant reviews. Conference abstracts and preprints (grey literature) were also searched.

### Eligibility Criteria

We have included randomized controlled trials that compared tirzepatide directly with semaglutide in adults. The studies had to report at least one efficacy outcome (absolute change in weight, proportion of participants who achieved a >10 percent weight loss, or change in HbA1c) or safety outcome (serious adverse event, discontinuation of treatment because of adverse event, nausea and vomiting, diarrhea, constipation, dyspepsia, decrease in appetite, injection site reaction etc). We excluded the non-randomized studies, letter to editor, case reports, editorials, and narrative reviews.

### Study Selection

Titles and abstracts were independently screened by two reviewers after which the shortlisted articles were subjected to full-text evaluation based on the eligibility criteria. The conflicts were settled by discussion; when it was not possible to reach consensus a third reviewer ruled.

### Data Extraction

Data were extracted using a standardised form. Recorded variables included first author, publication year, study design, sample size per arm, follow-up duration, and participant demographics variables. In the case of dichotomous outcomes, the number of events and the number of subjects per group were counted. In the case of continuous results, the mean, standard deviation, and the sample size of each group were assimilated. All the extracted data were verified by two reviewers.

### Quality Appraisal

The Cochrane Risk of Bias 2 (RoB 2) tool [12] was used to assess the risk of bias on each of the included trials and assesses five domains (randomisation process, non-adherence to intended interventions, missing outcome data, measurement of the outcome, and choice of the outcome) to determine the risk of bias. All the areas were rated as low risk, some concerns, or high risk. Assessments were made by two reviewers and disagreements were sorted out by the third reviewer.

### Statistical Analysis

Review manager version 5.4 was used for the analyses. In the case of dichotomous outcomes, the Mantel Henszel method was used to obtain risk ratios (RR) with 95% confidence interval (CI). In the case of continuous outcomes, the inverse-variance method was used to compute mean differences (MD) with 95% CI. The I^2^ statistic was used to determine the heterogeneity. A statistical significance of p < 0.05 was considered. Publication bias was not assessed due to limited no. of studies in this meta-analysis.

## Results

### Study Selection

**Figure 1:**
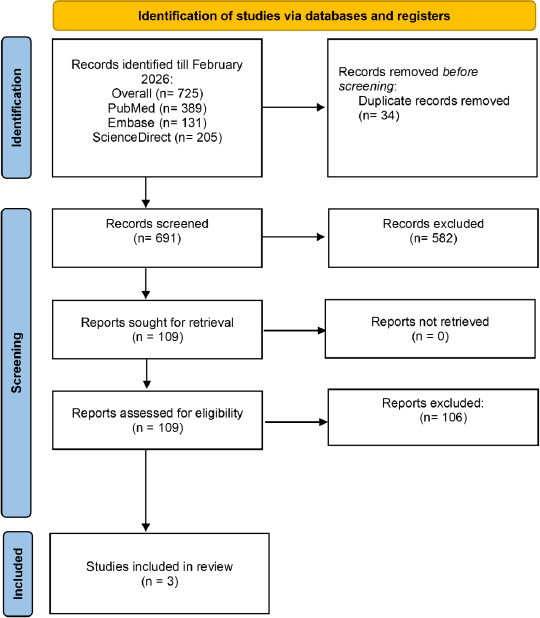
PRISMA flowchart

The PRISMA flowchart (Figure 1) illustrates the study selection process. A comprehensive electronic literature search conducted until February 2026 across 3 databases identified 725 records (PubMed: n = 389; Embase: n = 131; ScienceDirect: n = 205). After removal of 34 duplicate records, 691 titles and abstracts were screened, of which 581 were excluded. Full-text reports of the remaining 109 records were sought for retrieval and assessed for eligibility. Of these, 106 reports were excluded, resulting in the inclusion of 3 studies [8,9,23] in the systematic review and meta-analysis.

### Characteristics of Included Studies

The three included trials were published between 2021 and 2025. Two (Aronne 2025, Frías 2021) were open-label and one (Heise 2022) was double-blind. Follow-up ranged from 28 to 72 weeks. The pooled sample comprised 2,717 participants (1,828 tirzepatide; 889 semaglutide). Mean age ranged from 45.0 to 63.7 years. Female representation ranged from 23% to 64.7%. Mean baseline BMI ranged from 30.8 to 39.4 kg/m^2^, and mean HbA1c from 7.70% to 8.29%.. A brief overview of the study characteristics is given in Table 1.

### Clinical Outcomes

**Figure 2.**
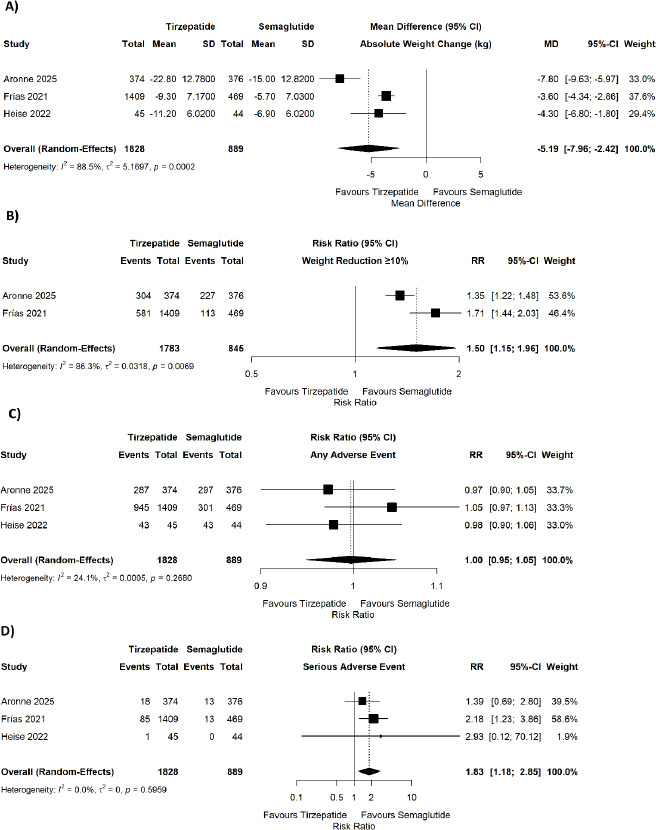
Forest plots showing the comparison of tirzepatide versus semaglutide for (A) absolute weight change (kg), (B) weight reduction ≥10%, (C) any adverse event, and (D) serious adverse event.

In the included studies, tirzepatide was associated with a significantly greater absolute weight reduction compared to semaglutide (pooled MD = −5.19, 95% CI −7.96 to −2.42; p = 0.0002; I^2^ = 88.5%) (Fig. 2A) and a significantly higher likelihood of achieving ≥10% body weight reduction (pooled RR = 1.50, 95% CI 1.15–1.96; p = 0.0069; I^2^ = 86.3%) (Fig. 2B).

**Figure 3.**
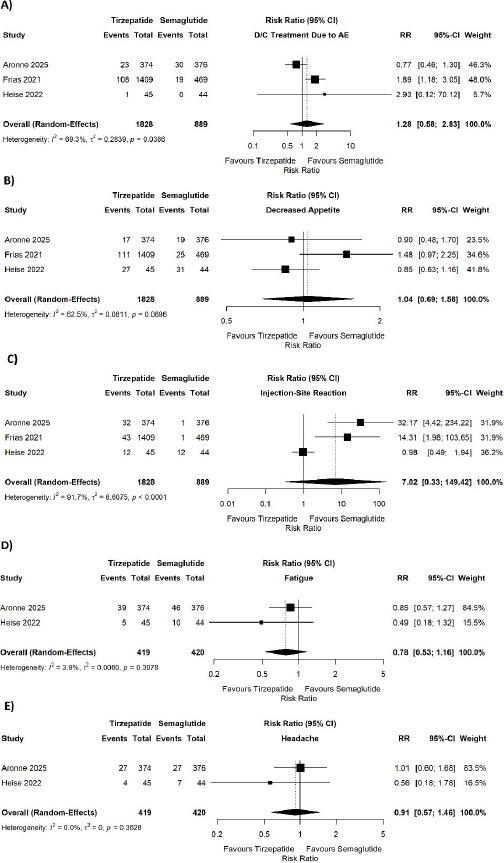
Forest plots showing the comparison of tirzepatide versus semaglutide for (A) discontinuation of treatment due to adverse events, (B) decreased appetite, (C) injection-site reaction, (D) fatigue, and (E) headache.

**Figure 4.**
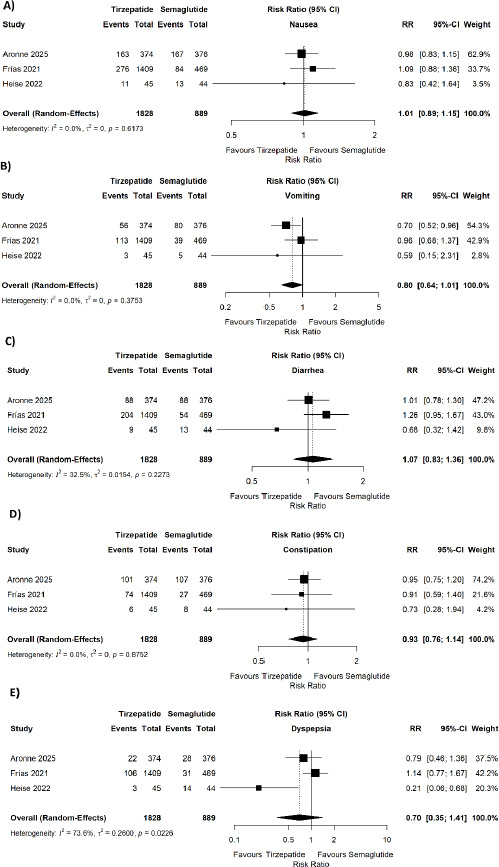
Forest plots showing the comparison of tirzepatide versus semaglutide for (A) nausea, (B) vomiting, (C) diarrhea, (D) constipation, and (E) dyspepsia.

However, tirzepatide was associated with a significantly higher risk of serious adverse events (pooled RR = 1.83, 95% CI 1.18–2.85; p = 0.007; I^2^ = 0%) (Fig. 2D). No significant difference was observed in discontinuation of treatment due to adverse events (pooled RR = 1.28, 95% CI 0.58–2.83; p = 0.54; I^2^ = 69.3%) (Fig. 3A), decreased appetite (pooled RR = 1.04, 95% CI 0.69–1.58; p = 0.0696; I^2^ = 62.5%) (Fig. 3B), injection-site reaction (pooled RR = 7.02, 95% CI 0.33–149.42; p < 0.0001; I^2^ = 91.7%) (Fig. 3C), fatigue (pooled RR = 0.78, 95% CI 0.53–1.16; p = 0.3078; I^2^ = 3.9%) (Fig. 3D), or headache (pooled RR = 0.91, 95% CI 0.57–1.46; p = 0.3628; I^2^ = 0%) (Fig. 3E). No significant differences were found for nausea (pooled RR = 1.01, 95% CI 0.89–1.15; p = 0.6173; I^2^ = 0%) (Fig. 4A), vomiting (pooled RR = 0.80, 95% CI 0.64–1.01; p = 0.3753; I^2^ = 0%) (Fig. 4B), diarrhea (pooled RR = 1.07, 95% CI 0.83–1.36; p = 0.2273; I^2^ = 32.5%) (Fig. 4C), constipation (pooled RR = 0.93, 95% CI 0.76–1.14; p = 0.8752; I^2^ = 0%) (Fig. 4D), dyspepsia (pooled RR = 0.70, 95% CI 0.35–1.41; p = 0.0226; I^2^ = 73.6%) (Fig. 4E), These results are shown in Figures 2–4. Sensitivity analysis was also done for the outcomes showing quite a lot of heterogeneity and we found that after removal of Aronne et al. 2025, the heterogeneity decreased. The I^2^ became 52% for the absolute weight reduction and 54% for the ≥10% body weight reduction.

### Quality assessment

**Figure 5.**
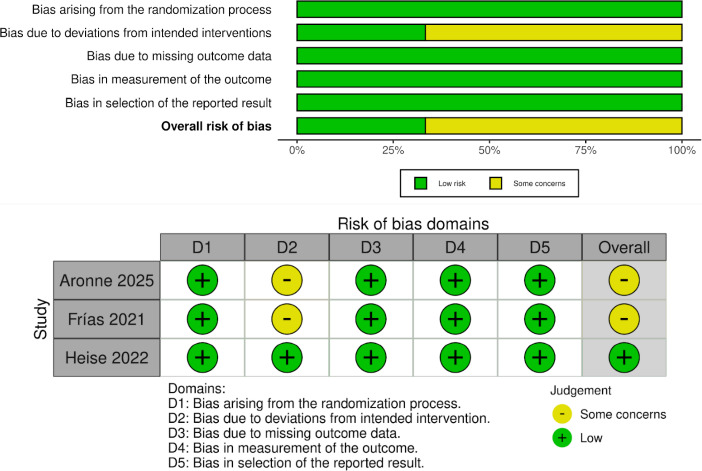
Summary and traffic light plot of risk of bias assessment using RoB 2.0 tool.

The risk of bias of the included studies was assessed using the RoB 2.0 tool (Figure 5). Two open-label trials (Aronne 2025 and Frías 2021) were judged to have some concerns in Domain 2 because of the absence of blinding. The double-blind trial (Heise 2022) was rated at low risk of bias across all five domains. Consequently, the overall risk of bias was rated as some concerns for two studies and low risk for one study.

## Discussion

This meta-analysis synthesized evidence of three head-to-head RCT comparing tirzepatide and semaglutide in terms of efficacy and safety. The main conclusions are that tirzepatide is much more effective in weight reduction and more likely to reach clinically relevant weight loss targets, and the two drugs have an otherwise similar adverse event profile except that more serious adverse events are observed with tirzepatide.

Tirzepatide has a dual receptor pharmacology that accounts for the weight loss benefit of this drug. As an imbalanced agonist, tirzepatide activates both GIP and GLP-1 receptors, but binds to the GIP receptor selectively over the GLP-1 receptor [13]. In mice, preclinical studies have demonstrated that co activation of GIP and GLP-1 receptors elevates POMC neuronal firing in the hypothalamic arcuate nucleus compared to either pathway alone and it also cause appetite suppression [14]. This two-central process is probably why tirzepatide is more effective in reducing body weight than selective GLP-1 RAs. Karagiannis et al. (2024) in a network meta-analysis of 28 RCTs found that tirzepatide 15 mg was the most effective treatment in both reducing HbA1c levels and reducing weight, with body weight reductions of 9.57 kg relative to placebo versus 4.97 kg with semaglutide 2.0 mg in patients with T2DM [15]. This observation is validated by our analysis, which is limited to direct comparisons, that indicates a difference of about 5 kg, in favour of tirzepatide, when pooled data is considered.

This amount of extra weight loss is clinically significant. The ADA Standards of Care of 2023 state that weight loss of more than 10% of the body can be disease-modifying and improve or cure the condition of obstructive sleep apnoea, T2DM and hypertension [16]. In our pooled analysis, tirzepatide-treated participants were half as likely to cross the ≥10% threshold as compared with semaglutide. A recent systematic review by Wen et al. (2025) including both RCT and observational data on direct tirzepatide-semaglutide comparisons also reported similar findings, that is, tirzepatide is always associated with greater weight loss, but with a higher overall adverse event rate [17]. Our individual analysis of the 14 separate types of adverse events contributes to their study by demonstrating that this general difference is not reflected in meaningful increases in most individual safety endpoints.

The very high incidence of serious adverse events with tirzepatide should be interpreted with care. The biggest sample study that we have used in our analysis was the SURPASS-2 trial, which was carried out in the COVID-19 pandemic, with the original researchers commenting that five deaths in the tirzepatide arms were subject to confounding or caused by COVID-19 [8]. This time confounder could have disproportionately inflated the number of serious adverse events. A pharmacovigilance study by Tobaiqy and Elkout (2024) with EudraVigilance database did not determine a significantly different serious adverse event profile of tirzepatide and semaglutide among reported cases [18]. It is yet to be determined whether this signal is maintained with larger samples and extended follow-up by current trials like SURMOUNT-MMO, a cardiovascular outcomes trial of tirzepatide in obese adults with known cardiovascular disease [19].

The fact that no significant difference in the number of individual gastrointestinal adverse events occurred between the two agents can be explained by the fact that these events are a class effect of incretin-based therapy. They are mediated via delayed gastric emptying and central nausea pathways and are most likely to be seen especially in dose escalation [20]. All three trials involved gradual dose-titration schedules, which probably blunted the severity and occurrence of early gastrointestinal adverse events in both arms. This pattern also was found to be similar in a study conducted by Nauck and Muller (2022) [21]. No significant difference in gastrointestinal tolerability between the two drugs was also reported in a post-marketing comparative safety review that was conducted by Singh et al. (2025) [22].

No comment can be made on the data on the injection-site reaction. Heise 2022 reported equal rates whereas the other two trials were almost exclusive to the tirzepatide arm. Such discrepancies are possibly due to the difference in injection device, formulation, or reporting rates and not a pharmacological action, and the convergence of the estimates should not be considered side effects as there are few investing studies [23].

### Clinical Implications

When clinicians are deciding between these two agents, our results imply that tirzepatide has a distinct benefit in terms of magnitude of weight reduction and the probability of achieving clinically significant weight reduction levels. Nevertheless, even possibly confounded, the serious adverse event warning is a wake-up call to patient selection and monitoring. In established cardiovascular disease patients, semaglutide now has more compelling evidence of a 20% relative risk reduction in major adverse cardiovascular events [23] and this should be incorporated into clinical practice until cardiovascular outcomes outcomes of tirzepatide are known.

### Limitations

There are a number of limitations that must be recognized. Firstly, the number of RCTs that satisfied our inclusion criteria was only three, which restricts the statistical power of possible rare outcomes and constrains the generalisability of our results. Second, two of the three trials were open-label, which could introduce the risk of introducting assessment and performance bias. Third, there was no access to patient-level data, which could not allow us to perform subgroup analyses based on dose, diabetes status, and BMI category. Lastly, this discussion is restricted to the short- and medium-term outcomes; it is not yet clear how weight loss is sustained in the long term and the cardiovascular safety profile of tirzepatide compared to semaglutide.

## Conclusion

This meta-analysis of head-to-head RCTs demonstrates that tirzepatide produces significantly greater weight loss than semaglutide, with a comparable gastrointestinal safety profile. The higher rate of serious adverse events with tirzepatide requires further investigation in larger and longer-term studies. Both agents remain effective options for weight management and glycaemic control, and the choice between them should be individualised based on treatment goals, comorbidity profile, and available cardiovascular outcome evidence.

## Figures and Tables

**Table 1:** Characteristics of the Included Studies

Author, Year	Study Design	Follow-up (weeks)	Tirzepatide (n)	Semaglutide (n)	Total (n)	Country
**Aronne et al. 2025 [9]**	Open-label RCT	72	374	376	750	United States and Puerto Rico
**Heise et al. 2022 [23]**	Double-blind RCT	28	45	44	89	Germany
**Frías et al. 2021 [8]**	Open-label RCT	40	1409	469	1878	United States, Argentina, Australia, Brazil, Canada, Israel, Mexico, and the United Kingdom

**Table 2.** Baseline Demographic and Clinical Characteristics of Participants

Variable	Aronne et al. 2025 [9]		Heise et al. 2022 [23]		Frías et al. 2021 [8]	
	Tirzepatide	Semaglutide	Tirzepatide	Semaglutide	Tirzepatide	Semaglutide
**Age (years, mean ± SD)**	45.0 ± 12.9	44.4 ± 12.7	61.1 ± 7.1	63.7 ± 5.9	56.4 ± 10.3	56.9 ± 10.8
**Female (%)**	64.7	64.6	31.0	23.0	53.4	52.0
**Male (%)**	35.3	35.4	69.0	77.0	46.6	48.0
**Body weight (kg, mean ± SD)**	112.7 ± 24.8	113.4 ± 26.3	94.2 ± 14.0	92.7 ± 14.0	93.7 ± 22.1	93.7 ± 21.1
**BMI (kg/m^2^, mean ± SD)**	39.4 ± 7.4	39.4 ± 7.7	31.3 ± 5.0	30.8 ± 3.8	34.2 ± 6.8	34.2 ± 7.2
**Waist circumference (cm, mean ± SD)**	117.7 ± 16.1	118.8 ± 17.6	NA	NA	109.4 ± 15.5	109.0 ± 14.9
**SBP (mmHg, mean ± SD)**	NA	NA	137.2 ± 13.0	135.9 ± 14.5	130.8 ± 14.1	130.0 ± 13.0
**HbA1c (%, mean ± SD)**	NA	NA	7.83 ± 0.72	7.70 ± 0.60	8.29 ± 1.03	8.25 ± 1.01
**Fasting glucose (mg/dL, mean ± SD)**	NA	NA	139.3 ± 30.2	128.6 ± 25.0	173.5 ± 52.0	171.4 ± 49.8
